# The impact of guidance counselling on gender segregation: Major choice and persistence in higher education. An experimental study

**DOI:** 10.3389/fsoc.2023.1154138

**Published:** 2023-04-06

**Authors:** Melinda Erdmann, Juliana Schneider, Irena Pietrzyk, Marita Jacob, Marcel Helbig

**Affiliations:** ^1^President's Research Group, Berlin Social Science Center, Berlin, Germany; ^2^Institute of Sociology and Social Psychology, Faculty of Management, Economics and Social Sciences, University of Cologne, Cologne, Germany; ^3^Department of Educational Decisions and Processes, Migration, Returns to Education, Leibniz Institute for Educational Trajectories, Bamberg, Germany

**Keywords:** gender-atypical major, students' persistence, higher education, gender segregation in higher education, person-major fit, switching major, study satisfaction, dropout

## Abstract

Gender segregation in higher education is considered one of the main drivers of persistent economic gender inequality. Yet, though there has been considerable research identifying and describing the underlying mechanisms that cause gendered educational choices in higher education, little is known about how gender segregation in higher education could be changed. Accordingly, this article aims to determine the potential of educational interventions during high school to foster gender desegregation in higher education. We focused on two different processes that contribute to gender segregation in majors among higher education graduates: first, the selection into specific majors and, second, the selection out of specific majors. We investigated whether an intensive counselling programme leads to more gender-atypical choices among high-school graduates and examined whether intensive counselling supports several indicators of students' persistence in gender-atypical majors. Based on data from an experimental study of a counselling programme for German high-school students (*N* = 625), we estimated the programme's effect with linear probability models and intention-to-treat analysis. Our results show that high-school graduates are more likely to choose a gender-atypical major if they have received intensive counselling. This applies more to men than to women. In addition, the programme improved some persistence indicators for students in gender-atypical majors. Although we found a significant programme effect only for perceived person–major fit and student satisfaction, the coefficients of all aspects of students' persistence show a trend indicating that the programme was beneficial for students in gender-atypical majors. As experimental studies can also be affected by various types of bias, we performed several robustness checks. All analyses indicated stable results. In conclusion, we suggest that intensive counselling programmes have the potential to reduce gender segregation in higher education. More students were motivated to choose a gender-atypical major, and different aspects of student persistence were supported by the programme for students in gender-atypical majors.

## 1. Introduction

Gender segregation in higher education is considered one of the main drivers of persistent economic gender inequality. Many studies have shown that the gender pay gap can be partially explained by gender differences in major choices (Brown and Corcoran, 1997; Bobbitt-Zeher, 2007; Leuze and Strauß, 2014). Horizontal gender segregation has also been seen to produce many negative consequences. A diverse workforce, however, increases economic productivity as demonstrated by a number of researchers (van Knippenberg et al., 2004; Ali et al., 2011; Post and Byron, 2015). Furthermore, gender segregation of labour reproduces gender stereotypes, maintains unequal pay of different occupations, and may even perpetuate gender power relations in society (Reskin, 1993; Correll, 2004). For this reason, there has been considerable research identifying and describing the underlying mechanisms that cause gendered educational choices in higher education. Therein, educational institutions have been identified as one of the main factors contributing to gender segregation and, ultimately, to segregation in the labour market (e.g., Smyth, 2005; Bobbitt-Zeher, 2007). In light of these findings, various educational interventions have been implemented at different educational stages to combat inequalities. Most interventions have tried to foster desegregation by encouraging young women to enrol in male-dominated subjects or majors. However, little is known about whether these educational interventions lead to gender desegregation in completed majors among higher education graduates.

Previous research on gender segregation in higher education has focused either on the gendered choices of majors or on the gender composition among graduates of specific majors. However, it is worthwhile investigating the gender segregation among higher education graduates as a result of both: gendered selection into specific majors and non-completion of specific majors, i.e., gendered selection out of specific majors.

First, women more often choose majors in human-centred fields, whereas men more often choose majors in technical, math-intensive and things-oriented fields (Barone, 2011). The process of selection into specific majors is characterised by institutional barriers such as university admission requirements and by individual (gender-specific) decisions. Because institutional admissions restrictions are *per se* gender neutral, only the gender-specific perception of these barriers and individual gender-specific choices of majors lead to gender segregation in majors. Second, students with gender-atypical majors in higher education show higher dropout rates. This pattern of leaving gender-atypical fields has been called the “revolving doors” phenomenon (e.g., Jacobs, 1989; Meyer and Mantinger, 2021). Thus far, only a few interventional studies have been conducted to determine whether these two selection processes might be changed.

For the gendered selection into specific majors, several recent experimental studies have explored interventions intended to reduce gendered major choices in higher education. These studies mainly investigated short interventions such as information sessions in school, focusing especially on the effect of additional information about labour-market prospects (e.g., Barone et al., 2019; Finger et al., 2020; Pekkala Kerr et al., 2020). The results are mixed and do not support the idea that a generalizable consistent lack of information about rewards is the main driver of gendered choices in majors. Piepenburg and Fervers (2021) examined a more comprehensive intervention and found that it positively affected students' intentions to enrol in gender-atypical majors and in majors other than those that were well-known. Nevertheless, the authors suggest that further research is needed to determine whether comprehensive interventions support desegregation in higher education (Piepenburg and Fervers, 2021). Regarding the selection out of specific majors, we are not aware of any intervention studies investigating how students with gender-atypical majors might be encouraged to persist to complete their majors.

Against this backdrop, we investigate educational interventions for high-school students, evaluating their potential to foster gender desegregation in higher education. The two selection processes noted earlier give rise to our two research questions. First, will an intensive counselling programme promote the choice of gender-atypical majors among high-school graduates? Second, will such a programme support the persistence of students in gender-atypical majors? In addressing these questions, we used data from an experimental study on the effect of an intensive counselling programme on German high-school students (*N* = 625). As part of the intervention, students were counselled regarding their career and post-secondary education options and supported in implementing their decision. The overarching goal of the counselling programme was to reduce social inequality in university enrolment as well as to improve the fit between individual interests and post-secondary educational pathways. The programme provided personalised long-term support through individual meetings with qualified counsellors. The direction of the personal counselling was determined by the student's individual needs, questions and insecurities. The use of experimental data enhanced the internal validity of our causal conclusions about the impact of intensive counselling on gendered major choices and on students persisting in the chosen major.

Our experimental study contributes to previous research on gender desegregation in higher education in several important respects. First, the counselling programme we investigated was a more comprehensive intervention, as it was tailored to each student's individual needs, questions and insecurities. Second, we estimated the programme's effect on gender desegregation by applying an elaborate experimental design. Third, unlike previous research on gender segregation in higher education, we addressed two relevant outcomes that jointly contribute to gender segregation: selection into and selection out of specific majors. Thus, we explored different ways in which desegregation might be promoted.

Our analysis showed that an intensive counselling programme reduces gender segregation in higher education by affecting both selection processes. The programme increased enrolment in gender-atypical majors, especially for men. It also positively affected various predictors of persistence for students enrolled in gender-atypical majors. Based on these results, we point out three observations that can ground policy recommendations and future research. First, gender segregation in higher education seems to result from two different selection processes, which occur at different points in the educational trajectory, and each can be addressed by an educational intervention. This may be effective in counteracting gender segregation and, consequently, gender inequality in the labour market. Second, whereas current political measures mostly address the scarcity of women in male-dominated fields, our results suggest that gender segregation can also be counteracted through measures that encourage both women and men to enrol in gender-atypical majors. Third, even though gendered interests develop early in life and gender-specific choices are often set before secondary education begins, our results indicate that interventions for high-school seniors may still contribute to desegregation in higher education.

The remainder of this article is structured as follows. In Section 2, we provide an overview of the state of research on gender segregation in higher education and introduce our theoretical framework. In Section 3, we describe our experimental data and methodological strategy for answering the research questions. We present our study's results in Section 4, beginning with the effect of an intensive counselling programme on the choice of gender-specific majors followed by results on students' persistence in gender-atypical majors. In Section 5, we describe several robustness checks undertaken to address some methodological challenges. In the final section, we discuss implications for research and policymaking as well as the methodological limitations of our analysis.

## 2. Previous research and theoretical considerations

A considerable amount of theoretical and empirical research exists on gender segregation in higher education. However, research has predominantly aimed to understand and describe the different processes that lead to gender segregation in education (for a literature review, see Yazilitas et al., 2013; Wang and Degol, 2017). Research on interventions that foster desegregation in higher education is scarce. As we focus on two selection processes that can result in gender segregation, and as they operate at two different stages of the higher educational trajectory, we briefly discuss the existing (experimental) research on gender segregation in higher education in two steps. First, we discuss the research on gendered choices of majors in higher education; second, we review the research on persistence among students who have made gender-atypical choices. Based on these discussions, we elaborate on why an intensive and individual counselling programme may contribute to fewer gendered major choices and to students persisting in the chosen gender-atypical major.

### 2.1. Gendered educational choices

The phenomenon of gender differences in major choices in higher education has been well-researched in sociology. To date, however, only a few sociological and economic studies have examined specific interventions targeting high-school graduates transitioning from high school to higher education regarding gendered choices of major, and even fewer have used an experimental design. Below, we examine existing experimental studies to inform our theoretical considerations and hypotheses.

#### 2.1.1. Previous research

A few recent sociological and economical studies have addressed interventions and their effects on young people's major choices. The treatments in these studies range from short information sessions provided in the classroom (Barone et al., 2019; Finger et al., 2020) to additional information presented during counselling that was a mandatory component of the school curriculum (Pekkala Kerr et al., 2020) to a full-day counselling workshop provided by professional university counsellors (Piepenburg and Fervers, 2021). In the first three studies—Barone et al. (2019) in Italy, Finger et al. (2020) in Germany and Pekkala Kerr et al. (2020) in Finland—the information sessions took place in classrooms. These sessions informed high-school seniors about returns, costs, and funding options in higher and vocational education. In these studies, the researchers assumed that high-school students have inaccurate perceptions of the economic returns associated with a given field of study—perceptions that additional information could perhaps rectify. The targeted outcome of the studies was the choice of a more rewarding field of study. Barone et al. (2019) found a treatment effect, but it only applied to women: only women were redirected to more rewarding fields after treatment. In contrast, Finger et al. (2020) found an effect only on men's applications to, and enrolment in, more rewarding fields. In the Finnish context, Pekkala Kerr et al. (2020) could not find any treatment effect, neither for young women nor for young men. It remains unclear whether the differences in empirical findings result from slightly different settings and treatment durations or from country differences, say, in the labour market. Whatever the cause, these mixed results do not support the idea that a generalisable consistent lack of information about rewards is the main driver of gendered major choices. A slightly more comprehensive counselling intervention was studied by Piepenburg and Fervers (2021). The treatment consisted of a 1-day group workshop offered by professional university counsellors, which included a self-assessment whereby students tested both their cognitive and non-cognitive skills as well as their vocational interests; they also received feedback on majors that might fit their individual interests and abilities. The authors found a positive effect on high-school students' intentions to enrol in gender-atypical majors (Piepenburg and Fervers, 2021).

To summarise, the aforementioned studies of gender desegregation in higher education leave us with some open questions. First, though, in some countries, providing information about different majors seems to affect students' choices, these studies concentrated on field-specific rewards. Although male-dominated fields are better compensated on average than female-dominated fields, there is still variation between gender-typical fields; for example, education and medicine are both female-dominated fields, and medicine brings higher earnings. Hence, the extent to which this type of counselling may contribute to gender desegregation in higher education remains an open question. Second, the study that examined gender-atypical choices considered enrolment intentions without providing separate analyses by gender. Hence, it remains unclear whether actual enrolment in gender-atypical majors is affected and whether the effect is heterogeneous by gender.

The next section takes a theoretical perspective, discussing a student's choice of a gender-atypical major as a deviation from gendered norms that may require particular support and advice. We pay particular attention to potential differences in men's and women's receptivity to counselling.

#### 2.1.2. Theoretical considerations

Previous sociological research has often explained gendered educational choices in higher education using two dominant theoretical frameworks. The first theoretical framework is socialisation theory, which proposes that girls and boys develop gender-specific vocational interests and career aspirations based on gender stereotypes (e.g., Marini et al., 1996; Charles and Bradley, 2002; Correll, 2004). Parents, peers and further significant others affect girls' and boys' behaviour and preferences, and peers of the same gender are especially influential role models in cultivating these gender stereotypes (Eccles and Hoffman, 1984; Marini and Brinton, 1984). The second framework is rational choice theory, which suggests that gender differences in chosen fields result from gender-specific evaluation of costs, benefits and probabilities of success (e.g., Gabay-Egozi et al., 2015; Barone et al., 2017; Lörz and Mühleck, 2019). Despite their focus on theories of information perception (such as dual process theory), all of the abovementioned studies refer in some manner to at least one of these two frameworks. Furthermore, the two frameworks complement each other in terms of temporal path dependency. Thus, from a life course perspective, the first theoretical framework explains how young people develop gendered educational and occupational aspirations, whereas the second one explains how they choose between different educational options.

Some developmental theories of occupational aspiration and socio-psychological theories also address these two aspects: the development of occupational aspiration and the determinants of young people's occupational decisions. For instance, according to Gottfredson (1981, 2002), young people develop their occupational aspirations based on a cognitive map of suitable occupations, which they adjust in light of perceived constraints. Gottfredson (1981), in her theory of circumscription and compromise, described the individual process for developing occupational aspirations by invoking two interrelated mechanisms. The first mechanism describes “the progressive and usually permanent circumscriptions of occupational preferences according to one's developing self-concept” (Gottfredson, 1981, p. 545). The second mechanism describes the way young people make compromises based on their perceptions of the opportunities for realising their choices. Similarly, in her achievement-related choice model, Eccles (1994, p. 590) explained how “gender roles likely influence educational and vocational choices, in part, through their impact on individuals' perceptions of the field of available options, as well as through their impact on expectations and subjective task value.” According to the theories of Gottfredson and Eccles, at a very early stage of development, young people begin to develop their self-concepts, chart occupations on a cognitive map and formulate preferences by sex type. Thus, sex types and gender norms have a very powerful effect on a person's consideration of their different educational and vocational options. This is because sex is a central aspect of the self-concept and serves as a more obvious cue than other aspects, such as social status (Gottfredson, 1981). Young people often unconsciously reject gender-atypical options without evaluating them because they have assimilated culturally defined gender roles (Eccles, 1994). Furthermore, even if young people aspired to gender-atypical occupations that reflected their interests, they would sacrifice these interests, which are less visible characteristics, before making a choice that conflicted with gender or social norms (Gottfredson, 1981).

Given this strong but unreflective reluctance to aspire to (and enrol in) gender-atypical majors, counselling may encourage students to consider gender-atypical occupations by helping students expand their range of possibilities by introducing them to occupations that were excluded from their cognitive maps at a very early stage. Furthermore, counselling may also support students who already aspire to a gender-atypical major. Such students have not yet compromised their interests to meet gender-specific norms, and external advice could perhaps help them realise their bold aspirations. Therefore, educational interventions informing students about various vocational options and encouraging high-school seniors to follow their interests may increase gender-atypical choices.

For three different reasons, the short interventions described in the previous section, all of which focused on the costs and benefits of different occupational options, are unlikely to change young people's perceptions of occupational options. First, they do not provide enough new experiences or modifications of students' social environment to change young people's perceptions of occupational options—i.e., to alter what Gottfredson (2002) called the cognitive map. Second, parents, peers and other role models, as already mentioned, often influence gendered occupational aspirations (Eccles and Hoffman, 1984; Marini and Brinton, 1984). Unlike individualised, intensive counselling programmes, short interventions do not give young people the chance to build close relationships with counsellors or meet others who could function as role models. Third, the abovementioned short interventions did not consider either the high-school student's individual interests or the match between individual interests and educational options. Yet, according to both the theoretical framework of Gottfredson (2002) and the empirical research by Piepenburg and Fervers (2021), the match between interests and educational options is essential to breaking down choices driven by gender norms. Therefore, short interventions focusing solely on non-personalised information may fail to support young men and women in leaving “beaten” gendered paths. Assuming that an intensive (individual and long-term) counselling programme could provide such support and encouragement, *we expect the counselling programme to increase the number of students choosing a gender-atypical major (H1a)*.

Moreover, given the theory and empirical evidence on gender differences in the development of occupational aspirations, we expect to find a heterogeneous effect by gender. In addition to gender type, an individual's self-concept includes their (future) social position in society (Gottfredson, 1981). Whereas, for women, abandoning gender norms by choosing a gender-atypical major in many cases results in higher earnings and social status, many men who choose a female-dominated field earn less and have a lower social status than they would have had otherwise. Hence, men may be more restricted in their choices of gender-atypical majors than women. As a consequence, counselling might be more effective for women. Thus, *we expect the counselling programme's effect on gender-atypical major choice to be more pronounced for young women than men (H1b)*.

### 2.2. Students' persistence in gender-atypical majors

In addition to the gendered choice of major, a reduced persistence of students in gender-atypical study programmes may contribute to gender segregation in higher education. Given the lack of studies on interventions regarding academic success in gender-atypical fields, we briefly summarise research on the more general issue of academic success in gender-atypical fields. We then elaborate on whether and in what respect counselling could help students overcome obstacles.

#### 2.2.1. Previous research

Several studies have examined the persistence and dropout of students with gender-atypical major choices. Most of them found lower persistence and higher dropout rates for students with gender-atypical major choices compared to students with gender-balanced or gender-typical choices (e.g., Meyer and Strauß, 2019). However, mixed results have been obtained for the relationship between the gender composition of fields and student persistence. Some studies have shown higher dropout rates for females in male-dominated fields (Meyer and Strauß, 2019, for Germany), some have found higher dropout rates for males in female-dominated fields (Severiens and Ten Dam, 2012, for the Netherlands) and others have found higher dropout rates in male-dominated fields for both genders (Mastekaasa and Smeby, 2008, for Norway). These mixed results are not surprising given the different country contexts, research designs, operationalisations of dropouts and reference groups. Although the patterns of gender-specific dropout vary, the gender-specific reasons for leaving male- or female-dominated fields are less diverse. Female students who have left male-dominated fields usually did so because they lacked confidence in their abilities or had become disappointed and lost motivation, whereas male students who have left male-dominated fields usually failed due to a lack of ability (Severiens and Ten Dam, 2012; Meyer and Mantinger, 2021). Men in female-dominated fields are observed less frequently, but, if they did drop out more often than women, the predominant reasons are perceived prejudice and a lack of peer support (Severiens and Ten Dam, 2012).

In summary, students in gender-atypical fields are more likely to drop out than other students. This seems primarily due to false expectations about their abilities and requirements, but also due to disappointment and a lack of peer support during demanding phases of their studies. It seems plausible that counselling provided before enrolment to explain the requirements of specific fields and target a student's motivation and resilience could potentially increase success, especially in gender-atypical fields. However, we could not find any study that investigated an intervention to foster persistence in gender-atypical majors.

#### 2.2.2. Theoretical considerations

Researchers have proposed different theoretical explanations for lower persistence within gender-atypical majors, and, admittedly, some of them could not be directly addressed by individual counselling. This is the case for mechanisms located on the institutional or societal level, such as a “chilly climate” in a male-dominated field that discourages women (Hall and Sander, 1982; Lee and Mccabe, 2021) and the devaluation of tasks in female-dominated fields (see the devaluation theory of England, 1992) that discourages men. However, many researchers also refer to Tinto (1975) theoretical model of students' departure, highlighting the importance of the individual's academic and social integration, which intensive counselling could help support even before students enter higher education. Academic integration refers to the student's grade performance and intellectual development, whereas social integration refers to the student's interactions with peer groups and faculty members. Both integration processes affect commitment, which, in turn, influences persistence in or dropout from a study programme (Tinto, 1975, p. 95). Empirical research indicates the importance of both types of integration for students persisting in their study programmes by minorities, including students with gender-atypical major choices (Tinto, 1997; Severiens and Ten Dam, 2012; Meyer and Strauß, 2019).

Both academic and social integration in higher education could be supported both before and after enrolment. In a broad sense, academic integration implies that students are well-aware of their chosen field's formal (and informal) requirements. Such information about female-dominated fields is presumably less available to young men and vice versa during the aspirational stage and decision process, as described in the previous section. Counsellors could help close this information gap. In addition, professional encouragement for students to trust their abilities may help them succeed in gender-atypical fields, keeping them from losing their self-confidence when they face obstacles. Social integration implies belonging to academic groups and interacting with other students and faculty members. A failure to integrate into a chosen gender-atypical field could provoke a loss of self-confidence and motivation. Counsellors might introduce students to future peers and university life by offering activities with students who share similar interests and faculty members. Such activities could strengthen social integration, thereby helping students to navigate the empirically observed obstacles that accompany a gender-atypical major choice. Furthermore, counsellors might also motivate students and help them make contacts during their studies. Hence*, we assume that the counselling programme increases persistence for students with gender-atypical major choices (H2)*.

## 3. Data and methods

Data were collected *via* an experimental study^1^ of a counselling programme offered in North Rhine-Westphalia, Germany's most populous state. Our study investigated the impact of intensive counselling on the educational choices of high-school students attending the academic track (for further information, see Pietrzyk et al., 2019).^2^ In this section, we first describe the research design and the intervention, then the sample and variables and, finally, our analytical strategy.

### 3.1. Research design and treatment

Our study combined a panel survey of high-school students with randomised counselling treatment. The study included a survey with several waves, beginning in the second-to-last year of high school (academic track in comprehensive schools: grade 12; Gymnasium: grade 11) and ending 3 years after high-school graduation. As the counselling programme targeted socio-structurally disadvantaged high-school students in particular, schools with relatively high proportions of socio-structurally disadvantaged students constituted the majority of the sample.^3^ A total of 42 schools agreed to participate in the study. In early 2018, the first wave was conducted using a paper–pencil survey in the classroom; this provided the baseline measurement for the randomised controlled trial (*n* = 1.776 students). Due to the counselling programme's limited capacity, only 31 schools were randomly selected for the experimental study. Within these schools, 1,404 students were randomly assigned in a 50/50 allocation ratio to experimental conditions (a control condition without counselling and a treatment condition with counselling). School affiliation (school identification) and the educational level of the parents served as a blocking variable in the randomisation. Blocking guarantees an equal distribution of important characteristics across experimental conditions; for example, it ensures that the number of students whose parents hold a higher education degree is the same in the control and treatment groups.

During the course of the study, students were interviewed at different stages of their educational trajectory through several additional online surveys. During the second wave, the participants were close to their high-school graduation date (early 2019). The third wave (late 2019) captured the first possible transition to a post-secondary education pathway. During the fourth wave (end of 2020), some participants were already in their second year of post-secondary education (for more information, see Pietrzyk et al., 2019).

The intensive counselling programme began with a one-on-one meeting between the student and a trained counsellor. To minimise the effort for students, the counsellors were sent to the schools. These counsellors, who typically have a higher education degree, had undergone specially designed training and were employed at the academic counselling service of a nearby university. The programme encouraged students to cooperate with counsellors in exploring their interests, future hopes and problems in choosing an educational pathway after graduation; specific initial concerns were also addressed as needed. Hence, the programme was tailored to each student's individual needs, questions and insecurities. In addition to the individual counselling sessions, the programme offered several activities that enabled participants to meet other students with similar interests, connect with professionals working in the careers pursued by students and take advantage of campus visits and referrals to other advisory services. The counselling programme's overarching goal was to dissociate high-school students' educational decisions from their social background by enhancing the fit between educational choices and individual capabilities and interests. The counsellors saw themselves as contact persons for all questions concerning post-school education, which could also include personal uncertainties. To ensure low-threshold accessibility and regular exchange, communication channels outside of meetings were also used in practise, such as exchanges *via* text messages. As noted, the programme began during senior classes in high school and continued, if necessary, for several years afterwards, and thus it was designed to provide long-term support. In this way, uncertainties arising during the post-secondary pathways could also be addressed.

### 3.2. Sample and variables

For the following analyses, we used the third and fourth wave of the survey data, which contained information on the students' educational pathways 0.5 and 1.5 years after graduating high school. As we were interested in the gendered major choice in higher education, we restricted our original experimental sample from the first wave (*n* = 1,145)^4^ to students who actually enrolled in university or a university of applied science (*n* = 772; control group *n* = 388, treatment group *n* = 384).^5^ Because the first year in higher education is considered the most critical phase (Trautwein and Bosse, 2017), our analyses used information from that year alone. More specifically, we used information from different time points (waves 3 and 4) because students started their study programmes at different times.^6^ Although we used two survey waves, each person is included in the analysis sample only once. By allowing for listwise deletion,^7^ our statistical analyses covered 625 cases of first-year students in higher education (control group *n* = 310; treatment group *n* = 315). Potential bias due to panel attrition, sample selection and the strategy to test and control for these biases are discussed in Section 5.

#### 3.2.1. Dependent variables

Our first dependent variable was whether students started a study programme in a gender-atypical major. In the survey, we asked our respondents about their majors by presenting a very detailed list of majors available in Germany. To identify the gender typicality of each major, we used German Federal Statistical Office data on first-semester students from 2017 to 2019 (Destatis, 2022),^8^ calculated the mean share of women for each major, and matched this information with the survey data. In line with recent research on gendered educational choices in higher education, engineering and information technology were strongly male-dominated majors, with men making up over 80% of the student population, whereas some areas of education and humanities were strongly female-dominated, with the share of women exceeding 80% (see the list of majors that are most strongly male- and female-dominated in Germany, Supplementary Table S.2). For the first descriptive illustration of the distribution, we created three categories for our first dependent variable. Hence, our first category, “gender-atypical major choice,” included all students who chose a major (first or second major) enrolling less than or equal to 40% of the respondent's gender.^9^ The opposite category, “gender-typical major choice,” included all students who chose a major enrolling more than or equal to 60% of the respondent's gender. The middle category, labelled “gender-balanced,” included the rest of the students. Given our focus on gender-atypical major choices in the final analyses, we used only binary coding for the primary dependent variable. Thus, we distinguished between a gender-atypical major choice coded as 1 if the share of the respondent's gender in the chosen major (first or second major) is less than or equal to 40% and a non-gender-atypical choice coded as 0 if the share of the respondent's gender in all chosen majors is higher than 40%.

Our second dependent variable addressed students' persistence. Since our analyses focused on an early stage in students' higher education careers, we could only use variables that served as proxies for students' persistence. To consider different aspects, we used students' reported perceived person–major fit, their overall satisfaction with their studies, their intention to switch majors, and their intention to drop out of higher education. All four aspects strongly predict a switch of major or a dropout of higher education (e.g., Eaton and Bean, 1995; Ertl et al., 2022; Meyer et al., 2022). We measured the perceived person–major fit based on the following question: “*Please indicate to what extent the following statements apply to you. I have chosen a major that suits me*.” The respondents could answer on a 7-point scale with 1 = “*not at all*” and 7 = “*very well*.”^10^ Students' level of satisfaction with their studies was measured with the question “*All in all: How satisfied are you with your studies overall?*” This could be answered on a 5-point scale with 1 = “*very unsatisfied*” and 5 = “*very satisfied*.” The intention to switch majors and the intention to drop out of higher education were measured with the questions “*How likely is it that you will change your major before completing your current degree?*” and “*How likely is it that you will drop out of your studies and do something completely different instead (e.g., start vocational training or work)?*” Both questions could be answered on a 5-point scale with 1 = “*very unlikely*” and 5 = “*very likely*.”

#### 3.2.2. Independent variables

Our main independent variable is the assignment to the treatment that was conducted beginning in the second-to-last year of high school. The variable is coded 0 for the control group and 1 for the group that was assigned to the counselling programme. Furthermore, in the first analyses, gender (0 = male, 1 = female) is used as a second independent variable to identify whether we find a heterogenous effect of the programme on gender-atypical choice.

#### 3.2.3. Controls

Given our focus on the effect of the intensive counselling programme and the experimental framework, we calculated parsimonious models with a small number of variables in the first place. In these models, we only controlled for the waves, parental education and the schools. Since the data were collected over a period of 1 year (0.5 years and 1.5 years after graduation), students could have been exposed to the programme for a different length of time before entering higher education. Hence, we included the survey wave (0 = 3rd wave and 1 = 4th wave) as a control variable. Furthermore, the data were clustered in schools. Because we used parental education and school identification as blocking variables during the randomisation, we included these variables as controls (school-fixed effects).

Table 1 shows the distribution of the dependent and independent variables by programme assignment with visible differences between the control and treatment groups for some of the dependent variables and small differences for the additional independent variables of parental education and initial school performance. The distribution of the first dependent variable shows that gender-atypical major choice is a rare phenomenon in our sample, with only 9% of students in the control group and 18% of students in the treatment group choosing a gender-atypical major.^11^ This small number of cases in the category of interest limited our possibilities to conduct detailed analyses in two ways. First, we could not change the cut-off for the definition of a gender-atypical major choice to test whether our results are robust if we use a more rigid cut-off. This is because with a more rigid cut-off the number of students with a gender-atypical choice would decrease. Second, we are not able to conduct stratified analyses that address the group of students with a gender-atypical choice. Therefore, separate analyses by gender were only feasible for testing our first two hypotheses (H1a and H1b).

**Table 1:** Descriptive statistics of the dependent and independent variables by programme assignment for the analytic sample.

	**CG**		**TG**	
	**Mean**	***n***	**Mean**	***n***
**Dependent variables**				
Gender-atypical major	0.09	310	0.18	315
Person-major fit	5.61	310	5.59	315
Satisfaction	3.46	310	3.45	315
Intention to switch majors	2.05	310	2.05	315
Intention to drop out of HE	1.73	310	1.66	315
**Independent variable**				
Gender (women)	0.59	310	0.59	315
**Control variable**				
Parents' education (HE degree)	0.52	310	0.46	315
Initial academic performance W1^a^	9.60	301	9.74	298
Wave (4^th^ wave)	0.31	310	0.33	315

a This variable is used as a control only in a robustness check. The initial academic performance was measured as the average grade of a 15-point grading scale out of seven different subjects (German, mathematics, English, physics, biology, history and social science). CG, control group; TG, treatment group.

### 3.3. Analytical strategy

Regarding the experimental design of the study, all forthcoming analyses followed the intention-to-treat strategy. This strategy considers participants' random assignment to the experimental conditions rather than their actual programme participation. As not all participants complied with the assignment (e.g., not all students invited to the programme met with the counsellor and vice versa), actual participation might not have been random. This could result in biassed estimation if actual participation was used as the independent variable. By using an intention-to-treat strategy in both groups, students who did not comply with the assignment (non-compliers) were also included. Because non-compliance also occurs in the programme's everyday practise, estimating the programme effect in this way mirrors the effect under real-world conditions (Hollis and Campbell, 1999).

The first central question in this article was whether gendered educational choices differ between students according to whether they were assigned to the counselling programme. We calculated the programme's effect on gender-atypical major choice by applying two different linear probability models. In the first model, the overall effect for all students was calculated. In the second model, we additionally calculated the interaction between gender and assignment to the programme. This enabled us to estimate gender-specific heterogeneous programme effects on major choice.

The second central question of our study asked whether assignment to an intensive counselling programme impacts the persistence of students in gender-atypical majors. We addressed this issue by estimating two different regression models. In the first model, we estimated the overall effect of the programme assignment on persistence, and, in the second model, we included an interaction term between assignment and gender-atypical major choice.

## 4. Results

**Figure 1:**
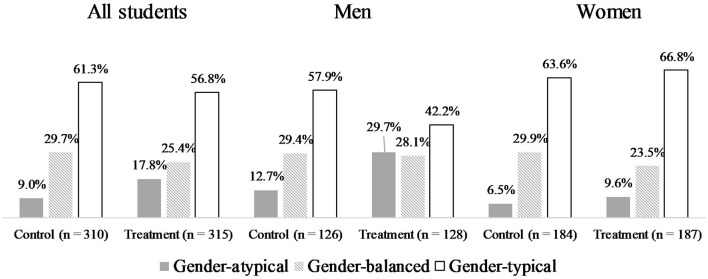
Gender composition within major by programme assignment for all students and by gender.

As a first step, we present the descriptive results. These results show the distribution of three different categories of gender composition within the chosen major (gender-atypical, gender-balanced and gender-typical) for students in the treatment and control groups and by gender (see Figure 1).

Concerning gender-atypical major choice, we already see notable differences between the control and treatment groups for all students (9.0% vs. 17.8%), but the separate distribution by gender reveals that these differences are primarily driven by male program participants (12.7% vs. 29.7%). Regarding this descriptive result, the programme markedly increased enrolment in a gender-atypical major. To see whether our sample differs from the overall population of first-semester students, we also calculated the distribution using Federal Statistical Office data on first-semester students (see Supplementary Figure S.1). A comparison with the control group of our sample showed that the patterns were similar. In our sample, without controlling for any confounders, the counselling programme had a positive effect on choosing a gender-atypical major, especially for men. Furthermore, when we observe the distributions, we see a gender-differentiated pattern between the control and treatment groups. For men, there was less difference between the treatment and control groups in the proportion of students choosing a gender-balanced major than there was for women. Although no conclusions can be drawn about the flow of students from one category to the other based on programme assignment, the distributions show that, among men in the treatment group, a higher proportion of students in gender-atypical majors is accompanied by a lower proportion of students with gender-typical choices. For women, the treatment group shows a slightly reduced proportion of gender-balanced major choices in favour of both gender-typical and gender-atypical choices, as compared to the control group. Overall, for women, the programme did not lead to many notable differences, whereas, for men, gender-atypical choices were more frequent and gender-typical choices less frequent among individuals assigned to the treatment group.

As a second step, we calculated two linear probability models with a binary coded dependent variable, “gender-atypical major choice,” to verify the descriptive results and to test our two first hypotheses. In Table 2, the first model (*Model 1a*) reveals an overall effect of programme assignment of 8.8 percentage points. Students who were assigned to the programme were significantly more likely to choose a gender-atypical major than students in the control group. This result verifies our first hypothesis (*H1a*), which expected the programme to increase the number of students making a gender-atypical choice. The results of the second model (*Model 1b*) indicate a strong negative interaction between the programme effect and being female. Thus, the programme effect is significantly less pronounced for women than for men. To illustrate this gender-specific programme effect in detail, we calculated the conditional average treatment effects with *p*-values for women and men based on the regression of Model 1b. As the results of Model 1b already suggest, the programme influenced gender-atypical major choice among men to a large extent—approximately 16 percentage points—whereas it had only a very small and non-significant effect on women's choice of major in terms of gender typicality (see Supplementary Table S.4: men ATE = 0.164, *p* = 0.001; women ATE = 0.033, *p* = 0.269). Even if these results indicated a gender-heterogenous programme effect, that would not support our second hypothesis (*H1b*). Contrary to our expectation that the programme would foster the choice of gender-atypical majors more among women, it in fact supported gender-atypical choices among men. Additionally, we calculated the same models with the share of women in majors (metric outcome). The results showed the same pattern (see Supplementary Table S.5).

**Table 2:** Results of the linear probability models on gender-atypical major.

	**Gender-atypical major**	
	**Model 1a**	**Model 1b**
Programme (assigned = 1)	0.088^***^	0.164^***^
	(0.028)	(0.051)
Gender (women = 1)		−0.062^*^
		(0.036)
Interaction (assigned^*^women)		−0.131^**^
		(0.060)
Parents' education (HE degree = 1)	0.030	0.019
	(0.030)	(0.029)
Wave (4^th^ wave = 1)	−0.065^**^	−0.038
	(0.029)	(0.029)
Constant	0.045	0.093
	(0.076)	(0.091)
*N*	625	625
Adj. *R*^2^	0.002	0.040

Robust standard errors in parentheses;

*** *p* < 0.01,

** *p* < 0.05,

* *p* < 0.1; both models with school-fixed effects.

Furthermore, we were interested in whether the negative association between gender-atypical choice and student persistence in higher education can be mitigated by an intensive counselling programme. Hence, the next analyses addressed different aspects of students' persistence. Table 3 shows the results for the perceived person–major fit, respondents' overall satisfaction with the study programme, their intention to switch majors and their intention to drop out of higher education. In Table 3, the second model for each outcome provides information for the main question, i.e., whether choosing a gender-atypical major was negatively associated with these aspects and whether we could find effect heterogeneity between students with and without a gender-atypical major.

**Table 3:** Results of the linear regression models with variables of students' persistence.

	**Person–major fit**		**Satisfaction**		**Intention to switch majors**		**Intention to drop out**	
	**Model 2a**	**Model 2b**	**Model 3a**	**Model 3b**	**Model 4a**	**Model 4b**	**Model 5a**	**Model 5b**
Programme (assigned = 1)	−0.025	−0.116	0.009	−0.063	0.002	0.023	−0.070	−0.019
	(0.106)	(0.111)	(0.091)	(0.097)	(0.099)	(0.104)	(0.083)	(0.091)
Gender typicality (atypical = 1)		−0.685^**^		−0.320		0.486^*^		0.137
		(0.333)		(0.258)		(0.287)		(0.226)
Interaction (assigned^*^atypical)		0.840^**^		0.559^*^		−0.352		−0.351
		(0.393)		(0.310)		(0.354)		(0.259)
Gender (women = 1)	0.007	−0.002	0.009	0.020	0.030	0.060	−0.109	−0.124
	(0.115)	(0.120)	(0.097)	(0.098)	(0.108)	(0.110)	(0.087)	(0.090)
Parents' education (HE degree = 1)	0.198^*^	0.189^*^	0.119	0.110	−0.129	−0.130	−0.014	−0.007
	(0.110)	(0.109)	(0.095)	(0.095)	(0.106)	(0.106)	(0.087)	(0.088)
Wave (4^th^ wave = 1)	0.130	0.122	−0.058	−0.058	0.042	0.053	0.141	0.139
	(0.112)	(0.112)	(0.104)	(0.104)	(0.111)	(0.111)	(0.091)	(0.092)
Constant	5.151^***^	5.188^***^	3.033^***^	3.039^***^	2.107^***^	2.063^***^	1.510^***^	1.515^***^
	(0.425)	(0.430)	(0.261)	(0.267)	(0.366)	(0.373)	(0.241)	(0.240)
*N*	*625*	*625*	*625*	*625*	*625*	*625*	*625*	*625*
Adj. *R*^2^	0.006	0.015	0.017	0.020	0.003	0.007	0.058	0.058

Robust standard errors in parentheses;

*** *p* < 0.01,

** *p* < 0.05,

* *p* < 0.1; all models with school-fixed effects.

For all four dependent variables, no overall effect was found (see *Model 2a/3a/4a/5a*). Furthermore, we could not observe any significant main effects of the programme in the models that included gender typicality and the interaction effects (see *Model 2b/3b/4b/5b*). Hence, we can conclude that the programme did not support students' persistence over all—that is, when all students are analysed simultaneously.

In line with previous German empirical studies and our reasoning, we found that a gender-atypical choice of major was negatively associated with all of the considered aspects of students' persistence. Hence, choosing a gender-atypical major tended to result in lower perceived person–major fit, lower satisfaction with the study programme, higher intention to switch majors and higher dropout intentions. For instance, choosing a gender-atypical major was associated with a worse person–major fit, as indicated by the significant negative coefficients in Model 2b (*b* = −0.685).

The interaction between programme assignment and gender typicality indicated whether these negative associations could be mitigated by the programme. For perceived person–major fit (*Model 2b*: 0.840, *p* = 0.033) and study satisfaction (*Model 3b*: 0.559, *p* = 0.072), we found significant interaction terms. This indicated that the programme effect on these outcomes was heterogeneous regarding the gender typicality of the chosen major. For students with a gender-atypical major choice, the programme had notable positive effects. To further illustrate the effect size for persons with a gender-atypical major choice and persons without one, we calculated the predicted margins and the conditional average treatment effects by gender typicality of the major based on the regression models for all outcomes (see Supplementary Table S.6). For the person–major fit and study satisfaction, we found the same pattern. For persons with a gender-atypical major choice, the programme had a positive effect on the perceived person–major fit and study satisfaction (see Supplementary Table S.6: person–major fit: ATE = 0.724, *p* = 0.054; satisfaction: ATE = 0.496, *p* = 0.091). Regarding the 7-point scale for the person–major fit and the 5-point scale for satisfaction, these results indicate that the intervention had a pronounced effect. For persons without a gender-atypical major choice, there was no programme effect (see Supplementary Table S.6: person–major fit: ATE = −0.116, *p* = 0.298; satisfaction: ATE = −0.063, *p* = 0.515). Remarkably, the programme seems not only to reduce the negative association between gender-atypical choice and both person–major fit and study satisfaction but also to fully compensate for the negative consequences of gender-atypical choices. In Model 2b, for example, the interaction terms were higher than the negative coefficients of the main effects of gender typicality. Thus, person–major fit and study satisfaction among persons with a gender-atypical choice in the treatment group reached the same level as those among persons without a gender-atypical major in the treatment group (see Supplementary Table S.6).

Based on this mitigating effect for two outcomes (perceived person–major fit and study satisfaction), we might assume that the intensive counselling programme helped students feel that they had found “the right place” with their gender-atypical majors. Nevertheless, the sizes of the coefficients of all aspects of students' persistence showed a trend indicating a heterogeneous effect based on gender atypicality. When considering the predicted margins and the conditional average treatment effects for all different dependent variables, we found sizable conditional average treatment effects for students with gender-atypical majors. Specifically, for study satisfaction, this is almost a half point on a 5-point scale as noted above (see Supplementary Table S.6: gender-atypical ATE = 0.496, *p* = 0.091). Although not all coefficients of the interaction between programme assignment and gender-atypical majors were significant, we saw that, descriptively, the programme had a positive effect on all different aspects of persistence for the students with gender-atypical majors. These results are in line with hypothesis *H2*.

In summary, our results show that an intensive counselling programme for high-school seniors fostered the choice of gender-atypical majors in higher education among men. Furthermore, the programme significantly enhanced both the perceived person–major fit and students' satisfaction for those with a gender-atypical major. No such effect can be seen for students without a gender-atypical major. Hence, we found that the programme promotes men's selection into gender-atypical majors and fosters two indicators of study persistence for male and female students in gender-atypical majors.

## 5. Robustness checks for post-treatment selection bias

To test whether our results are robust, we performed several further analyses. Although the purpose of an experimental design is to avoid an unequal distribution of confounding variables across experimental conditions, the presence of different types of biases cannot be ruled out *per se*. For our analysis sample, we identified three different sources of potential selection bias that could lead to different group compositions and thus biassed results.^12^

The first potential bias concerns the possibility of asymmetrical panel attrition. This may arise from different participation patterns for the surveys of students in the control and treatment groups. To rule out such unequal panel attrition, we considered several characteristics collected for baseline measurement. The differences between the experimental conditions for individuals who did not participate in the third and/or fourth wave were approximately the same as for individuals who did participate in those waves. Therefore, we found no evidence of significant asymmetrical panel attrition (see Supplementary Table S.8).

The second possible bias may arise from the exclusion of individual cases due to item nonresponse. A comparison between included and excluded cases showed asymmetrical differences between the control group and treatment group for three outcome variables (perceived fit, intention to switch and intention to drop out) and the initial academic performance at the first wave (see Supplementary Table S.9). To check whether the observed differences are signs of a significant selection bias in our sample, we calculated a linear probability model with the inclusion status (1 = included, 0 = excluded) as a dependent variable. We did not find any significant interaction between treatment assignment and the indicators of students' persistence (see Supplementary Table S.10). Third, selection by chosen post-secondary educational track may lead to a biassed sample, especially if the programme has a heterogenous effect on post-secondary educational choice. In our previous research on the programme's effect, we showed that the programme increases enrolment in higher education for persons with parents who did not graduate from higher education (Erdmann et al., 2022). Because the parents' education is an important blocking variable in the experimental design, we had already calculated all analyses taking parents' education into account. Furthermore, our previous research showed that the effects of the parents' education were heterogeneous by initial academic performance but not by other student characteristics. To consider the selection bias resulting from the heterogenous programme effect, we also calculated all models using students' initial academic performance in the first wave as a robustness check. Initial academic performance was measured as the average grade on a 15-point grading scale in seven different subjects (German, mathematics, English, physics, biology, history and social science). Again, the results showed the same pattern and the same significance (see Supplementary Tables S.11, S.12). Furthermore, the programme's positive effect on enrolment in gender-atypical fields of study could be driven by the increased enrolment in higher education of young people who aspired to a gender-atypical occupation in the first place. As a consequence, the programme's positive effect on enrolment in gender-atypical fields might be overestimated. This could be the case if former students who have gender-atypical occupational aspirations and who would otherwise have entered gender-atypical vocational training are more strongly induced by the programme to enrol in higher education. In this case, an increase in gender-atypical major choices will not be caused by changing students' aspirations to a gender-atypical field but, rather, by changing their chosen vertical educational path. Hence, we also ran analyses for persons in vocational training^13^ to determine whether this group shows an opposite pattern, which would indicate a selection bias by treatment and chosen track. The analysis suggests that there is no selection bias (see Supplementary Table S.13).

In addition to these potential selection biases, other factors may have compromised the internal validity of the causal effect estimates. These include the violation of the stable unit treatment value assumption (SUTVA), according to which the potential outcome of a study unit that participated in the measure does not affect the potential outcome of a study unit that did not participate in the measure (Imbens and Rubin, 2015). The SUTVA assumption can be influenced by spill-over effects, namely by possible friendships among the students. For instance, students who participated in the counselling programme might have influenced students from the control group, and, as a consequence, individuals from the control group might have been more likely to enrol in a gender-atypical major. Although we could not test this spill-over effect, we assume that, in the case of such effects, the estimated programme effect would be biassed downwards.

Furthermore, the estimate of the programme effect may be biassed by so-called non-compliance. Non-compliance occurs when students assigned to programme participation through randomisation do not participate in counselling and vice versa. This non-compliance with assignments becomes problematic when the non-compliance is non-random (Sagarin et al., 2014). In our analysis sample, 84.6% of participants conformed to the randomisation. However, 8% participated in counselling even though they were assigned to the control group, whereas 7.4% did not use counselling even though they were assigned to the treatment group. One approach described in the literature as the gold standard for dealing with non-compliance is the applied intention-to-treat analytic strategy (Sagarin et al., 2014). Another approach discussed is the analysis with an instrumental variable, which determines the potential effect that would be achieved with total compliance (Sagarin et al., 2014). Applying an instrumental variable in our models leads to a higher and more significant positive effect on enrolment in a gender-atypical major (see Supplementary Table S.14).

## 6. Discussion

Because gender segregation in higher education has many negative consequences, including gender-based income inequality (Brown and Corcoran, 1997; Bobbitt-Zeher, 2007; Leuze and Strauß, 2014), lower productivity (van Knippenberg et al., 2004; Ali et al., 2011; Post and Byron, 2015) and the reproduction of gender stereotypes and power relations (Reskin, 1993; Correll, 2004), promoting gender desegregation in higher education has significance for various aspects of gender equality. We assumed that guidance counselling may promote gender desegregation by influencing two distinct processes that constitute gender segregation in completed majors among higher education graduates: first, selection into specific majors and, second, selection out of specific majors. Regarding selection into specific majors, we expected that counselling might cause new occupations to emerge on a student's cognitive map of suitable occupations (Gottfredson, 1981). We further expected that it might support students' pursuit of occupations that were already present on their cognitive map, preventing them from compromising their occupational aspirations. Concerning selection out of specific majors, we assumed that counselling may support various aspects, including social or academic integration in gender-atypical majors (Tinto, 1975), which could transform into higher persistence.

In line with these theoretical considerations, we investigated whether counselling promotes the choice of gender-atypical majors and whether it helps students in gender-atypical majors to persist in their studies. Given the scarcity of research on whether educational interventions stimulate gender desegregation in higher education, our research on how gender desegregation can be supported by educational programmes constitutes a significant expansion. By evaluating an intensive counselling programme, we investigated a comprehensive educational intervention that could have a considerable influence on educational pathways. By applying an experimental design, we ensured a methodologically rigid approach. By addressing two relevant outcomes that jointly contribute to gender segregation among higher education graduates—selection into and selection out of specific majors—we explored different ways of how desegregation might be promoted. Taken together, these components rendered our investigation of the potential of educational interventions for gender desegregation both broad and methodologically neat.

Overall, we showed that the investigated counselling programme stimulated gender desegregation in two ways: it promoted gender-atypical major choices and fostered persistence in gender-atypical majors. First, we showed that the programme positively influenced enrolment in gender-atypical majors. This is in line with previous results on the effect of counselling on the intention to enrol in gender-atypical majors (Piepenburg and Fervers, 2021). More detailed analyses revealed that the programme's influence was particularly strong on men's major choices. For men, counselling fostered gender-atypical major choices by approximately 16 percentage points. Given that atypical major choices are relatively rare—in our sample's control group, a mere 13% of male students, approximately, chose gender-atypical majors—we consider the effect we found as being rather large; it suggests that atypical major choices more than doubled for men. However, we did not expect men's choices to be affected more strongly than women's; given the risk of social demotion for men, we assumed the programme would have a more pronounced impact on women's major choices. The fact that it had a greater influence on men's choices could perhaps derive from different causes, with one possible reason being the counsellors' features. Most counsellors have an academic background in female-dominated majors, such as education, psychology, social work, humanities and social sciences, and it is possible that they were especially enthusiastic about those majors, providing extensive information about them and their corresponding careers. Further, students may have perceived the counsellors as role models for studying these subjects. In general, if the counselling programme were broadly implemented, it would potentially lead to a much more frequent choice of atypical majors among men, to a reduction of horizontal gender inequalities and, in turn, to a noticeable mitigation of the negative effects of these inequalities. As a result, a broad implementation might affect not only men but also women in the long term. For one thing, a more balanced gender composition in formerly female-dominated fields could lead to a greater appreciation of these fields, which might be reflected in higher income. After all, it is not only the unequal gender composition in specific occupations but also their valuation that poses a social challenge. Furthermore, if men studied formerly female-dominated fields more frequently, that might help mitigate gender stereotypes, which could, in turn, have a positive effect on women's decisions.

Second, we observed that counselling positively affected some predictors of students' persistence in gender-atypical majors. The perceived fit between person and major and the level of satisfaction with the studies were both positively influenced by the programme among students enrolled in a gender-atypical major. This result suggests that counselling reduced the otherwise pronounced selection out of gender-atypical majors—a phenomenon resulting from various obstacles students face in gender-atypical fields of study. Regarding these outcomes once again, the effect sizes we observed were rather large, as the results yielded effects of around 0.7 on a 7-point scale and 0.5 on a 5-point scale. It is not easy to establish how exactly these effects translate into the actual completion of atypical majors. However, given the rather large effects on two indicators of persistence, it is likely that actual completion of gender-atypical majors was considerably promoted by the counselling programme, which, if scaled up, could potentially lead to noticeable gender desegregation and its positive consequences.

Overall, the programme had a positive effect on both selection into gender-atypical majors and selection out of gender-atypical majors to an important degree. Thus, counselling fostered gender desegregation to a significant extent in two different ways.

Our results are important because they encourage further research and can inform educational policies in a number of ways. First, when dealing with the impact of educational interventions on gender desegregation in higher education, we theoretically suggested not only focusing on the choice of major but also on persistence in gender-atypical majors. Our empirical results indicate that this theoretical perspective is useful; educational interventions have the potential to mitigate obstacles in gender-atypical majors and might thereby contribute to gender desegregation in higher education. In further research, it may be useful to expand the focus on persistence in gender-atypical majors when assessing the impact of interventions on gender desegregation. Second, we found that counselling about the choice of major might be particularly beneficial for men. This finding serves as a reminder that gender desegregation can be promoted from two sides. In addition to interventions supporting young women to aspire to enrol and persist in male-dominated majors, programmes addressing young men's educational careers can also foster gender desegregation in higher education. Although many interventions designed to promote gender desegregation are still targeted exclusively at young women, our results may stimulate a change of perspective in educational policies. Third, we find an educational intervention targeting high-school students in their final two years before graduation to be efficient for promoting gender desegregation. Gendered interests develop early in life (Gottfredson, 1981), and gender-specific choices are often set before secondary education. For that reason, some researchers have argued that interventions aiming at gender desegregation in higher education should be implemented early in the educational career (e.g., Mastekaasa and Smeby, 2008; Barone and Assirelli, 2020). However, our results show that an intensive intervention that begins at a later stage of the educational career can still promote gender desegregation in higher education, and, again, it can do so through two distinct modes of action: choice of major and persistence in gender-atypical majors. Further research should clarify whether and under which conditions an intervention's intensity can compensate for a late start.

Despite these implications for further research and policies, our study has some limitations. First, our analyses for the gender specificity of the programme effect were limited by the small sample size. The lack of statistical power may explain the low statistical significance of the programme effect on women, whereas a study with a larger sample size might examine whether the programme significantly increases women's gender-atypical choices. Furthermore, we could not break down our analysis of the intervention effect on the persistence of students with gender-atypical choices by gender. Thus, it remains unclear whether the positive effects for students in gender-atypical majors are attributable to both genders or only one. Both open questions should be answered in further research with a larger sample size. Second, instead of measuring real dropout from gender-atypical majors, we could use only predictors of persistence. Although the applied variables generally predict dropout quite well, further research should replicate our findings with a measure of real actions within higher education. Third, due to sampling with a focus on schools that were attended on average by socially disadvantaged students, the composition of social origin in our sample probably does not correspond to the social composition in all German high schools—even though our sample also included students from high social origins. Because research could find intersectionality between social origin and gender (e.g., van de Werfhorst, 2017), we assume that young people whose parents did not graduate from higher education are particularly likely to conform to gender-conforming behaviour. These students' more pronounced gender-conforming conduct may translate into lower responsiveness to educational programmes that aim at gender desegregation. If this is indeed the case, our results will be seen as conservative estimates of the programme effect on major choice and study persistence. However, further research should generally test whether our findings can be replicated with other samples in different national education systems. Fourth, we could not uncover the mechanisms that drive the investigated effects. Regarding the choice of major, for example, we were not able to indicate whether the programme changes the aspiration to a gender-atypical major and, respectively, to a gender-atypical occupation, or whether it “merely” fosters the realisation of a gender-atypical aspiration that already existed before programme participation.^14^ Stated in more theoretical terms, we cannot say whether the programme leads new occupations to emerge on an individual's cognitive map (Gottfredson, 1981) or whether it helps students pursue plans for occupations that were already present on their cognitive map. Regarding the programme effect on study persistence, possible mechanisms are even more diverse; counsellors may have supported various aspects, including social or academic integration (Tinto, 1975), which may have turned into higher persistence. Students may have been better prepared before they started higher education, or they may have met with their counsellors during their university studies.

Although we do not know precisely how the intensive counselling programme works, we nevertheless find that it fosters gender desegregation to a significant extent and in two distinct ways: by promoting the choice of gender-atypical majors and by supporting students' persistence in gender-atypical majors. We believe that our study provides important insights, and we accordingly hope it will stimulate research on educational interventions that aim at gender desegregation in higher education. We recommend the following three focuses on target group, starting point and mode of action: young men as targets of interventions, intensive programmes that begin in the years before high-school graduation and programmes that influence not only major choices but also persistence in gender-atypical majors. The latter is a way to promote gender desegregation in higher education that previous research on educational interventions has neglected. Against the background of our findings, all three focuses appear promising.

## Data availability statement

The datasets presented in this article are not readily available due to data protection regulations. Requests to access the anonymized datasets should be directed to the corresponding author.

## Ethics statement

The studies involving human participants were reviewed and approved by WZB Research Ethics Committee. Written informed consent from the participants' legal guardian/next of kin was not required to participate in this study in accordance with the national legislation and the institutional requirements.

## Author contributions

JS, ME, and IP contributed to the conception and design of the study. ME and IP were in charge of the data collection. JS organised the database and performed the statistical analysis. ME wrote the first draught of the manuscript. JS, IP, and MJ revised the manuscript. All authors contributed to reviewing the manuscript critically for important intellectual content, reading, and approving the submitted version.

## Footnotes

^1^The study was approved by the WZB Research Ethics Committee on 06 November 2017. The study is registered on AEA RCT Registry (https://www.socialscienceregistry.org/trials/2738).

^2^In the state of North Rhine-Westphalia, students mostly acquire the higher education entrance qualification (Abitur) in two different types of schools: Gymnasium and Gesamtschule (comprehensive school). The Abitur's curriculum is highly standardised and has only a few opportunities for specialisation in specific subjects. The subjects taken in the last two or three years of school have no relevance for the enrolment in specific fields of study in higher education. Thus, course selection in secondary school does not determine the later field of study (Jacob et al., 2020).

^3^The selection process used a school social index that is available for secondary schools in North-Rhine Westphalia (Schräpler and Jeworutzki, 2016).

^4^Due to panel attrition (*n* = 199) and the withdrawal by one school (*n* = 60) from the programme, the original experimental sample of the first wave (*n* = 1,404) was reduced by 259 cases. For an overview of the balance of covariates between treatment and control groups of the first wave sample is provided in the Supplementary (see Supplementary Table S.1).

^5^For an in-depth discussion of possible biases due to sample selection, panel attrition and item-nonresponse, see Section 5.

^6^For students who enrolled in winter semester 2019, we used the information of the third survey wave, and, for students who enrolled in the summer semester 2020 or the winter semester 2020, we used the fourth survey wave.

^7^We decided against imputation because most missing information in the initial sample concerned the dependent variable (gender typicality of major) or was due to panel attrition. For both reasons, imputation is not recommended (von Hippel, 2007; Young and Johnson, 2015).

^8^Due to changes of the share of women and men over time, we used only a time span of three years to adjust for extreme variation between different years.

^9^Recent research has shown that over the last decades, subjects in higher education became more gender-balanced or even more female-dominated (England, 2010; Hägglund and Lörz, 2020). Therefore, the probability to observe a male-dominated major choice decreased in recent decades. To account for this change, we use a comparably high share of students as a criterion for defining gender atypicality. A similar coding was also used by Piepenburg and Fervers (2021) and Alon and Gelbgiser (2011).

^10^In the version of the German questionnaire, the original scale was inverted.

^11^A detailed list of gender-atypical majors is provided in the Supplementary (see Supplementary Table S.3).

^12^For a systematic overview of the number of cases for each type of selection, see Supplementary Table S.7.

^13^To identify the gender typicality of each field of vocational training, we used data from the Federal Statistical Office on persons in the first year of vocational training from 2017 to 2019 (Destatis, 2018, 2019a,b,c, 2020, 2021).

^14^Because we did not have pre-treatment information on the gender specificity of major preferences, no analyses could be performed.
