# Supplementary material for: The impact of guidance counselling on gender segregation: Major choice and persistence in higher education. An experimental study
Source: Front Sociol. 2023 Apr 6;8:1154138. doi: 10.3389/fsoc.2023.1154138 (PMC10118044; doi:10.3389/fsoc.2023.1154138)
Supplement: Supplementary file 1 [file Data_Sheet_1.docx]

Supplementary Material

The impact of guidance counselling on gender segregation: Major choice and persistence in higher education. An experimental study

Melinda Erdmann^*^, Juliana Schneider, Irena Pietrzyk, Marita Jacob, Marcel Helbig

*** Correspondence:** Melinda Erdmann: melinda.erdmann@wzb.eu

# Supplementary Figures and Tables

## Supplementary Figures

Figure S.1. Gender composition of first semester within major for all students and by gender based on German Federal Statistical Office (Destatis) data, 2017-2019 (Cut-off ≥60). Source: Destatis (2022). Genesis-online Database: Only First-Semester Students 2017-2019

## Supplementary Tables

**Table S.1.** Distributions of selected characteristics in wave 1 by programme assignment

|  | **Control group** | **Treatment group** | **Diff.** |
| --- | --- | --- | --- |
| Initial academic performance W1 | 8.89 | 9.10 | -0.21 |
| N | 657 | 653 | 1310 |
| Initial intention to enroll in higher education | 3.62 | 3.65 | -0.03 |
| N | 687 | 675 | 1362 |
| *Parents’ education* |  |  |  |
| HE degree | 0.52 | 0.52 | 0.00 |
| No HE degree | 0.48 | 0.48 | 0.00 |
| N | 681 | 679 | 1360 |

**Table S.2.** List of the first 10 majors in higher education with the highest share of men or women first-semester students based on German Federal Statistical Office (Destatis) data, 2017-2019

| **Men** | | **Women** | |
| --- | --- | --- | --- |
| Subject | % | Subject | % |
| Automotive Engineering | 92.1 | Pedagogy of Early Childhood | 91.3 |
| Mechatronics | 89.4 | Linguistic and Cultural Studies | 87.8 |
| Mechanical Engineering | 87.4 | Interior Design | 87.2 |
| Aerospace Engineering | 84.9 | Elementary Education/Primary Education | 86.8 |
| Electrical Engineering/Electronics | 84.8 | Special Needs Education | 86.1 |
| Computer Engineering/Technical Informatics | 84.5 | Nutritional Science | 85.3 |
| Manufacturing/Production Engineering | 83.7 | Learning Area Science/Subject Teaching | 84.5 |
| Supply Engineering | 83.2 | Veterinary Medicine | 84.3 |
| Communication and Information Technology | 82.4 | Library Science | 84.0 |
| Wood Technology/Fiber Technology | 82.0 | Social Pedagogy | 83.5 |
| Source: Destatis (2022). Genesis-online Database: Only First-Semester Students 2017-2019; Note: Major with fewer than 150 students were excluded. | | | |

**Table S.3.** Gender-atypical major in the analysis sample

| **Men** | | **Women** | |
| --- | --- | --- | --- |
| Control group | Treatment group | Control group | Treatment group |
| Biochemistry | Administrative Science | Electrical Engineering and Information Technology | Civil Engineering/ Engineering |
| German Studies/German | Applied Science of Linguistics | Business Informatics | Business Administration |
| Health Sciences/  Health Management | Biology | Civil Engineering/ Engineering | Business Informatics |
| Japanese Studies | Communication Science/Public Relations | Computer Science | Computer Science |
| Law | English Studies/English | Industrial Engineering (Engineering focus) | Economics |
| Media Science | Financial Management | Mechanical Engineering | Electrical Engineering/ Electronics |
| Medicine (General Medicine) | German Studies/German | Physics | Industrial Engineering (Economics focus) |
| Non-medical Professions/Therapy | Graphic Design/Communication Design | Police/Constitutional Protection | Industrial Engineering (Engineering focus) |
| Psychology | Interdisciplinary Studies (Focus on Art, Art science) | Water Management | Mechanical Engineering |
| Social Work | Interdisciplinary Studies (Focus on Law, Economics, and Social Sciences) |  | Police/Constitutional Protection |
|  | Islamic Studies |  |  |
|  | Japanese Studies |  |  |
|  | Law |  |  |
|  | Media Economics/Media Management |  |  |
|  | Medicine (General Medicine) |  |  |
|  | Psychology |  |  |
|  | Social Science |  |  |
|  | Social Work |  |  |
|  | Tourism |  |  |
| n = 16 | n = 38 | n = 12 | n = 18 |

**Table S.4.** Conditional average treatment effects by gender based on model 1b

|  | **Men** | | | **Women** | | | **Gender diff.** |
| --- | --- | --- | --- | --- | --- | --- | --- |
| *Dependent variable* | Mean CG | Mean TG | ATE  (p-value) | Mean CG | Mean TG | ATE  (p-value) | ATE  (p-value) |
| atypical choice | 0.128 | 0.292 | 0.164*** (0.001) | 0.066 | 0.099 | 0.033  (0.269) | -0.131**  (0.029) |
| N |  |  |  |  |  |  | 625 |
| Notes: Robust standard errors in parentheses; *** p < 0.01, ** p < 0.05, * p < 0.1; all models with school-fixed effects | | | | | | | |

**Table S.5.** Results of the linear regression model on share of women in majors

|  | Share of women in majors | |
| --- | --- | --- |
|  | *Model 1a* | *Model 1b* |
| Programme (assigned = 1) | 0.034** | 0.064** |
|  | (0.016) | (0.025) |
| Gender (women = 1) |  | 0.204*** |
|  |  | (0.020) |
| Interaction (assigned*women) |  | -0.048 |
|  |  | (0.030) |
| Parents’ education (HE degree = 1) | -0.013 | -0.003 |
|  | (0.016) | (0.015) |
| Wave (4th wave = 1) | 0.114*** | 0.078*** |
|  | (0.017) | (0.015) |
| Constant | 0.523*** | 0.372*** |
|  | (0.055) | (0.047) |
| N | 625 | 625 |
| Adj. R2 | 0.073 | 0.258 |
| Notes: Robust standard errors in parentheses; *** p < 0.01, ** p < 0.05, * p < 0.1; all models with school fixed effects | | |

**Table S.6.** Conditional average treatment effects by gender typicality of major in higher education based on model 2b – 5b

|  | **Not gender-atypical** | | | **Gender-atypical** | | | | **Typicality diff.** | |  |  |
| --- | --- | --- | --- | --- | --- | --- | --- | --- | --- | --- | --- |
| *Dependent*  *variable* | Mean CG | Mean TG | ATE  (p-value) | | Mean CG | Mean TG | ATE  (p-value) | | ATE  (p-value) | |  |
| Person–major fit (perceived) | 5.68 | 5.56 | -0.116  (0.298) | | 4.99 | 5.72 | 0.724* (0.054) | | 0.840** (0.033) | |  |
| Satisfaction | 3.48 | 3.42 | -0.063  (0.515) | | 3.16 | 3.66 | 0.496* (0.091) | | 0.559*  (0.072) | |  |
| Intention to switch majors | 2.01 | 2.03 | 0.023  (0.827) | | 2.49 | 2.16 | -0.330 (0.329) | | -0.352 (0.320) | |  |
| Intention to drop out | 1.72 | 1.70 | -0.019  (0.833) | | 1.86 | 1.49 | -0.370 (0.126) | | -0.351 (0.176) | |  |
| N |  |  |  | |  |  |  | | 625 | |  |
| Notes: Robust standard errors in parentheses; *** p < 0.01, ** p < 0.05, * p < 0.1; all models with school fixed effects | | | | | | | | | | | |

**Table S.7.** Differences between treatment and control groups by exclusion

|  |  |  |  | *Excluded due to …* | | | |  |
| --- | --- | --- | --- | --- | --- | --- | --- | --- |
| Characteristics in W1 | **Total** | **Included** | **Excluded** | *Item non-response* | *Unit non-response* | *Not in HE* | | |
| N (Total) | 1344 | 625 | 719 | 147 | 199 | 373 | | |
| Treatment (TG) | 0.50 | 0.50 | 0.50 | 0.47 | 0.53 | 0.49 | | |
| N | 1344 | 625 | 719 | 147 | 199 | 373 | | |
| Parents’ education (HE degree) | 0.47 | 0.49 | 0.44 | 0.56 | 0.47 | 0.38 | | |
| N | 1318 | 625 | 693 | 142 | 182 | 369 | | |
| Gender (women) | 0.57 | 0.59 | 0.55 | 0.65 | 0.48 | 0.54 | | |
| N | 1343 | 625 | 718 | 147 | 198 | 373 | | |
| Initial academic performance | 8.97 | 9.67 | 8.33 | 8.87 | 8.63 | 7.98 | | |
| N | 1259 | 599 | 660 | 132 | 176 | 352 | | |
| Notes: The original intended experimental sample included 1,404 students. Due to the withdrawal of one school (n = 60) immediately before the implementation of the programme, our total pre-treatment sample was reduced to 1,344 students. Because of the randomisation at the individual level within the schools, we do not assume any selection bias resulting from the drop out of this school. | | | | | | |  |  |

**Table S.8.** Differences between control and treatment groups by panel attrition

|  | **No participation in W3 and/or W4** | | | **Participation in W3 and/or W4** | | |
| --- | --- | --- | --- | --- | --- | --- |
| Characteristics | *CG* | *TG* | *Diff.* | *CG* | *TG* | *Diff.* |
| Initial academic performance W1 | 8.35 | 8.86 | -0.51 | 8.95 | 9.10 | -0.15 |
| N (Total = 1259) | 82 | 94 |  | 552 | 531 |  |
| Initial intention to enroll in higher education W1 | 3.54 | 3.46 | 0.08 | 3.62 | 3.67 | -0.05 |
| N (Total = 1304) | 87 | 101 |  | 571 | 545 |  |
| Gender (women) | 0.53 | 0.45 | 0.08 | 0.58 | 0.58 | 0.00 |
| N (Total = 1343) | 93 | 105 |  | 579 | 566 |  |
| Parents’ education (HE degree) | 0.48 | 0.47 | 0.01 | 0.47 | 0.47 | 0.00 |
| N (Total = 1318) | 84 | 98 |  | 575 | 561 |  |
| Total (N = 1344) | 93 | 106 |  | 579 | 566 |  |
| Notes: CG: Control group; TG: Treatment group; Diff.: CG-TG; Total number of cases with valid values for each variable in parentheses. | | | | | | |

**Table S.9.** Differences between treatment and control groups by sample selection

|  | **Excluded** (n = 147) | | | **Included** (n = 625) | | |
| --- | --- | --- | --- | --- | --- | --- |
|  | *CG* | *TG* | *Diff.* | *CG* | *TG* | *Diff.* |
| Gender-atypical major | 0.20 | 0.00 | 0.20 | 0.09 | 0.18 | -0.09 |
| N (Total = 634) | 5 | 4 |  | 310 | 315 |  |
| Person–major fit (perceived) | 5.47 | 4.85 | 0.62 | 5.61 | 5.59 | 0.02 |
| N (Total = 686) | 34 | 27 |  | 310 | 315 |  |
| Satisfaction | 3.16 | 3.11 | 0.05 | 3.46 | 3.45 | 0.01 |
| N (Total = 684) | 32 | 27 |  | 310 | 315 |  |
| Intention to switch majors | 2.36 | 2.19 | 0.18 | 2.05 | 2.05 | -0.01 |
| N (Total = 685) | 33 | 27 |  | 310 | 315 |  |
| Intention to drop out of HE | 2.06 | 2.22 | -0.16 | 1.73 | 1.66 | 0.07 |
| N (Total = 685) | 33 | 27 |  | 310 | 315 |  |
| Gender (women) | 0.67 | 0.62 | 0.04 | 0.59 | 0.59 | 0.00 |
| N (Total = 772) | 78 | 69 |  | 310 | 315 |  |
| Parents’ education (HE degree) | 0.57 | 0.54 | 0.03 | 0.52 | 0.46 | 0.06 |
| N (Total = 767) | 77 | 65 |  | 310 | 315 |  |
| Initial academic performance W1 | 8.83 | 8.92 | -0.09 | 9.60 | 9.74 | -0.13 |
| N (Total = 731) | 71 | 61 |  | 301 | 298 |  |
| Total (N = 772) | 78 | 69 |  | 310 | 315 |  |
| Notes: CG: Control group; TG: Treatment group; Diff.: CG-TG; Total number of cases with valid values for each variable in parentheses. | | | | | | |

**Table S.10.** Series of linear regressions models: inclusion status on treatment, one covariate and their interaction

| **Interaction variable** | **Coefficient** | **P-value** |  |
| --- | --- | --- | --- |
| Person–major fit (perceived) | 0.026 | 0.139 |  |
| Satisfaction | 0.002 | 0.938 |  |
| Intention to switch majors | 0.011 | 0.533 |  |
| Intention to drop out | -0.014 | 0.563 |  |
| Initial academic performance W1 | -0.001 | 0.922 |  |
| Notes: Robust standard errors in parentheses; *** p < 0.01, ** p < 0.05, * p < 0.1; Coefficients and p-values refer to the interaction term; | | | |

**Table S.11.** Results of the linear probability models on gender-atypical major with additional control for initial academic performance

|  | Gender-atypical major | |
| --- | --- | --- |
|  | *Model 1a* | *Model 1b* |
| Programme (assigned = 1) | 0.083*** | 0.148*** |
|  | (0.029) | (0.053) |
| Gender (women = 1) |  | -0.061 |
|  |  | (0.038) |
| Interaction (assigned*women) |  | -0.111* |
|  |  | (0.062) |
| Parents’ education (HE degree = 1) | 0.031 | 0.022 |
|  | (0.031) | (0.030) |
| Initial academic performance W1 | -0.009 | -0.006 |
|  | (0.007) | (0.007) |
| Wave (4^th^ wave = 1) | -0.072** | -0.046 |
|  | (0.029) | (0.030) |
| Constant | 0.154 | 0.163 |
|  | (0.116) | (0.130) |
| *N* | 599 | 599 |
| Adj. R^2^ | -0.003 | 0.027 |
| Notes: Robust standard errors in parentheses; *** p < 0.01, ** p < 0.05, * p < 0.1; both models with school-fixed effects | | |

**Table S.12.** Results of linear regression models with variables of students’ persistence with additional control for initial academic performance

|  | Person–major fit (perceived) | | Satisfaction | | Intention to switch majors | | Intention to drop out | |
| --- | --- | --- | --- | --- | --- | --- | --- | --- |
|  | *Model 2a* | *Model 2b* | *Model 3a* | *Model 3b* | *Model 4a* | *Model 4b* | *Model 5a* | *Model 5b* |
| Programme | -0.023 | -0.150 | 0.015 | -0.079 | 0.000 | 0.039 | -0.034 | 0.027 |
| (assigned = 1) | (0.107) | (0.115) | (0.093) | (0.099) | (0.102) | (0.107) | (0.082) | (0.090) |
| Gender typicality |  | -0.662** |  | -0.289 |  | 0.469 |  | 0.105 |
| (atypical = 1) |  | (0.325) |  | (0.257) |  | (0.288) |  | (0.215) |
| Interaction |  | 1.027*** |  | 0.666** |  | -0.436 |  | -0.393 |
| (assigned*atypical) |  | (0.374) |  | (0.309) |  | (0.353) |  | (0.248) |
| Gender | -0.053 | -0.046 | -0.029 | -0.008 | 0.071 | 0.090 | -0.054 | -0.074 |
| (women = 1) | (0.115) | (0.121) | (0.099) | (0.100) | (0.109) | (0.111) | (0.085) | (0.087) |
| Parents’ education | 0.202* | 0.185 | 0.101 | 0.086 | -0.124 | -0.121 | 0.026 | 0.036 |
| (HE degree = 1) | (0.114) | (0.113) | (0.095) | (0.095) | (0.111) | (0.110) | (0.088) | (0.089) |
| Initial academic performance W1 | 0.101*** | 0.101*** | 0.061** | 0.062** | -0.073*** | -0.072*** | -0.132*** | -0.133*** |
|  | (0.028) | (0.027) | (0.024) | (0.024) | (0.025) | (0.025) | (0.021) | (0.021) |
| Wave | 0.131 | 0.131 | -0.030 | -0.024 | 0.032 | 0.042 | 0.089 | 0.082 |
| (4^th^ wave = 1) | (0.115) | (0.114) | (0.106) | (0.106) | (0.114) | (0.114) | (0.090) | (0.091) |
| Constant | 4.060*** | 4.077*** | 2.285*** | 2.267*** | 2.803*** | 2.756*** | 2.806*** | 2.831*** |
|  | (0.559) | (0.565) | (0.349) | (0.356) | (0.497) | (0.503) | (0.345) | (0.342) |
| *N* | 599 | 599 | 599 | 599 | 599 | 599 | 599 | 599 |
| Adj. R^2^ | 0.028 | 0.041 | 0.028 | 0.035 | 0.014 | 0.017 | 0.118 | 0.120 |
| Notes: Robust standard errors in parentheses; *** p < 0.01, ** p < 0.05, * p < 0.1; all models with school-fixed effects | | | | | | | | |

**Table S.13.** Results of the linear probability models on gender-atypical field of vocational training programme

|  | Gender-atypical field of vocational training programme | | | |
| --- | --- | --- | --- | --- |
|  | | *Model 1a* | *Model 1b* |  |
| Programme (assigned = 1 | | 0.014 | 0.012 |  |
|  | | (0.059) | (0.085) |  |
| Gender (women = 1) | |  | -0.037 |  |
|  | |  | (0.085) |  |
| Interaction (assigned*women) | |  | 0.001 |  |
|  | |  | (0.112) |  |
| Parents’ education (HE degree = 1) | | 0.111** | 0.105* |  |
|  | | (0.055) | (0.057) |  |
| Wave (4th wave = 1) | | -0.039 | -0.034 |  |
|  | | (0.056) | (0.058) |  |
| Constant | | 0.129 | 0.139 |  |
|  | | (0.202) | (0.207) |  |
| N | | 183 | 183 |  |
| Adj. R2 | | 0.029 | 0.019 |  |
| Notes: Robust standard errors in parentheses; *** p < 0.01, ** p < 0.05, * p < 0.1; both models with school-fixed effects, Source: Destatis (2018, 2019a, 2019b, 2019c, 2020, 2021) | | | | |

**Table S.14.** Results IV-analysis with linear probability model on gender-atypical major choice

|  | Gender-atypical major |  |
| --- | --- | --- |
|  | *Model 1b (IV-analysis)* |  |
| Programme (participated/assigned = 1) | 0.127*** |  |
|  | (0.039) |  |
| Parents’ education (HE degree = 1) | 0.038 |  |
|  | (0.030) |  |
| Wave (4^th^ wave = 1) | -0.064** |  |
|  | (0.028) |  |
| Constant | 0.004 |  |
|  | (0.077) |  |
| *N* | 625 |  |
| Adj. R^2^ | -0.021 |  |
| Notes: Robust standard errors in parentheses; *** p < 0.01, ** p < 0.05, * p < 0.1; model with school-fixed effects | | |
